# The Relationship Between Disability-Adjusted Life Years of Cataracts and Ambient Erythemal Ultraviolet Radiation in China

**DOI:** 10.2188/jea.JE20140017

**Published:** 2015-01-05

**Authors:** Min Zhu, Jiaming Yu, Qian Gao, Yang Wang, Liwen Hu, Yang Zheng, Fang Wang, Yang Liu

**Affiliations:** 1 School of Public Health, China Medical University, Shenyang, People’s Republic of China; 2 Ophthalmology Department, The Fourth Affiliated Hospital of China Medical University, Shenyang, People’s Republic of China

**Keywords:** cataract, UVR exposure, disease burden, DALYs

## Abstract

**Background:**

Cataracts are one of the major public health problems worldwide. Ultraviolet radiation (UVR) is one of the risk factors for cataract development. We analyzed the relationship between disability-adjusted life year (DALY) rates of cataracts and UVR exposure in China.

**Methods:**

DALY rates of cataracts and UVR exposure in 31 regions of China were calculated based on data from the Second China National Sample Survey on Disability and the United States’ National Aeronautics and Space Administration database. The relationship between the DALY rates of cataracts and UVR was estimated by Spearman rank correlation analysis and linear regression analysis.

**Results:**

The elderly (≥65 years) had higher DALY rates of cataracts than the whole population. The DALY rate of cataracts in the agricultural population was higher than that observed in the non-agricultural population. The DALY rates of cataracts were positively associated with UVR The DALY rates of cataracts in regions with higher UVR were higher than those in regions with lower UVR. An increase in the daily ambient erythemal UVR of 1000 J/m^2^ was associated with an increase in the DALY rates of cataracts by 92 DALYs/100 000 (*R*^2^ = 0.676) among the whole population, 34 DALYs/100 000 among the population <65 years old (*R*^2^ = 0.423), 607 DALYs/100 000 among the population aged 65–74 years (*R*^2^ = 0.617), and by 1342 DALYs/100 000 among the population ≥75 years old (*R*^2^ = 0.758).

**Conclusions:**

DALY rates of cataracts increased with increases in UVR exposure in 31 regions of China. Greater exposure to UVR increases the disease burden of cataracts in the whole population, especially in the elderly and among the agricultural population.

## INTRODUCTION

Cataract is one of the major public health problems in the world today. About 47% of cases of low vision and blindness are due to cataracts globally.^1^ Together with cancer, blindness is considered one of the most feared health conditions.^2^ China has the largest number of visually disabled people in the world, and cataracts are the leading cause of this visual disability in China, accounting for 60% of all visual disabilities.^3^ Most cataract visual disability is age-related. Among the cataract-disabled people in China, over 80% are aged 65 years or older.^3^

Exposure to ultraviolet radiation (UVR) from the sun is one of the risk factors for cataract development.^4^ An early study performed by Hollows and Moran found that Australian aborigines who live in areas of high UVR are more likely to develop cataracts than their counterparts living in areas of low UVR.^5^ Other researchers have observed that the amount of time spent in the sun is associated with the development of cataracts.^6^^–^^8^ In a previous report, we found that the disability prevalence of cataracts was positively associated with ambient erythemal UVR in 31 regions of China, and we speculated that regions with higher urbanization might have lower disability prevalence of cataracts because of a lower level of outdoor exposure.^9^ Visual disability is the most serious health outcome caused by cataracts, which worsens the quality of life of cataract patients and brings heavy disease burden to the patients. However, not much is known about the relationship between the disease burden of cataracts and UVR exposure.

Disability-adjusted life years (DALYs) are a commonly used metric to quantify disease burden. One DALY can be thought of as one lost year of “healthy” life, and the burden of disease on a population can be thought of as a measurement of the discrepancy between current health status and an ideal situation where everyone lives into old age, free of disease and disability.^10^ Compared with traditional indices, such as incidence and prevalence, DALYs have the advantage of quantifying the burden of living with the disability caused by a disease. WHO estimates that approximately 20% of the DALYs caused by cortical cataracts are due to UVR exposure.^11^ In the present study, we aimed to establish the relationship between UVR and DALYs of cataracts in China, which will aid in understanding and demonstrating the severity of cataract visual disability due to UVR exposure. As most of the cataract-disabled people were elderly, we calculated DALYs of cataracts in different age groups, including a population <65 years old, a population 65–74 years old, and a population ≥75 years old. In addition, we also calculated DALYs of cataracts separately for agricultural and non-agricultural populations, because agricultural populations tend to have higher UVR exposure than non-agricultural ones, on account of spending more time performing outdoor labor.

The epidemiological characteristics of cataracts and unique geographic conditions of China make this setting ideal to study the relationship between cataracts and ambient erythemal UVR. The Second China National Sample Survey on Disability was able to provide accurate data on people who were visually disabled due to cataract and was notably helpful for our calculations of DALY rates (DALYs/sample size) of cataracts in the 31 regions. From a geographic point of view, the latitude from the southernmost part to northernmost part of China spans nearly 50 degrees, and the altitudes of the western and eastern regions differ by nearly 4000 meters, providing China with an uncommonly wide gradient of UVR exposure. The above advantages make it possible to analyze the relationship between ambient erythemal UVR and DALY rates in China.

## METHODS

### Survey on disability

**Figure 1.**
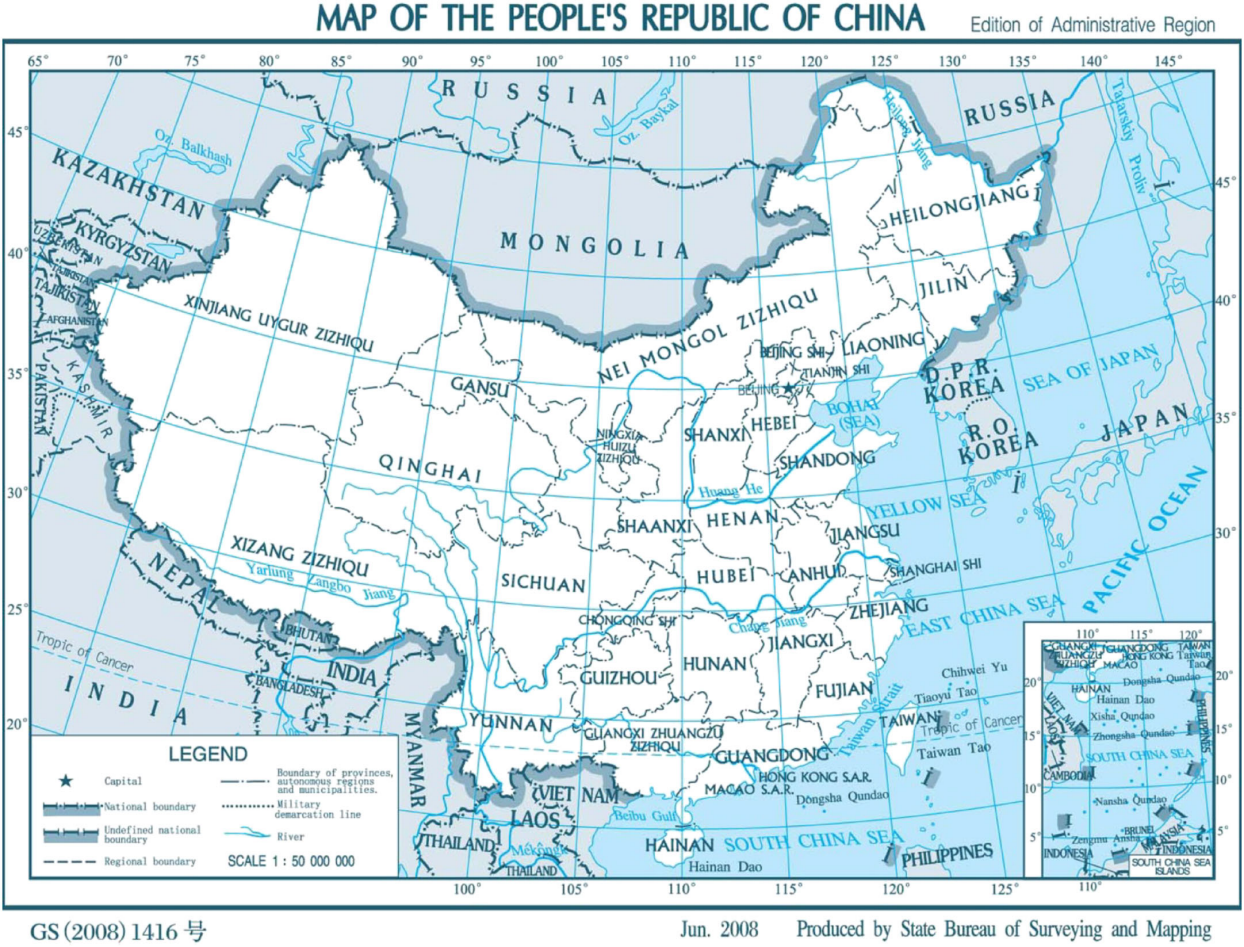
Map of China with the 31 regions included in the present study. Taiwan, Hong Kong, and Macau were not included in the Second China National Sample Survey on Disability and thus were not included in our analyses. Cited from the National Administration of Surveying, Mapping, and Geoinformation website, 2007. Standard map service. http://www.sbsm.gov.cn/article/zxbs/dtfw/ accessed 31 August 2011.

The data used to determine the DALYs of cataracts were obtained from the Second China National Sample Survey on Disability, which was a nationwide survey of disabled people carried out on April 1, 2006, in 31 regions, including 22 provinces, 5 autonomous regions, and 4 municipalities in mainland China.^3^ The geographic distribution of the 31 regions is shown in Figure 1. The survey used stratified, multi-phased, and cluster probability sampling designs, and sampling was conducted at four levels. The offices at the provincial level randomly selected 734 counties (cities or districts) based on economic development in each region. Each county then randomly selected 4 towns (with the exception of Beijing, Tianjin, and Shanghai, which selected more than 4 towns each) for a total of 2980 towns (townships or streets). Finally, each town and street randomly selected 2 communities, which were divided into a number of areas according to the size of their population. Each community randomly selected 1 survey area. The leading group of the Second China National Sample Survey on Disability was responsible for conducting the sampling. The number of sampling areas totaled 5964, with an average of 420 people living in each area. There were 2 526 145 people in 771 797 households investigated, and the sampling ratio (sample size/population size) was 1.93 per thousand.

The investigation was conducted in two stages. The first stage consisted of a household investigation, in which sampled households were asked to report the number of disabled individuals who suffer from abnormalities such as a loss in function (psychologically or physiologically) or anatomical structure of a certain organ and whether they had totally or only partially lost the ability to perform an activity in a way considered to be normal based on the guidelines provided in the screening table of the Second China National Sample Survey on Disability.^3^ The second stage involved the investigation of the identified disabled people, in which all candidates received medical examinations. The survey teams were responsible for visiting the sampled households and interviewing, screening, and assessing disabled people, thereby amassing detailed information on disabled people, such as the age of onset for disability and degree of disability. There were 738 survey teams organized, comprising more than 20 000 enumerators, nearly 6000 doctors of various specialties, more than 730 statisticians, and over 50 000 survey assistants.

The study assessed 161 479 disabled people, among which there were 18 085 people with visual disability caused by cataracts.^3^ In order to estimate DALYs, the number of incident cases during a particular time period was evaluated.^12^ In our study, patients who experienced the onset of cataract visual disability during 2003 to 2005 were selected. The final sample analyzed in this study was 6989 people, including 2526 people in 2003, 2446 people in 2004, and 2017 people in 2005. We separately calculated the DALY rates in these three years and used the mean of the DALY rates over the three years to stand for the average level of DALY rates. The final sample was divided into agricultural and non-agricultural populations according to the household register, resulting in an agricultural population of 5831 and a non-agricultural population of 1158.

### Ethics statement

The Second China National Sample Survey on Disability was a government-organized survey approved by the State Council of the People’s Republic of China. It was planned and coordinated by 16 ministries and agencies, including the National Bureau of Statistics, the Ministry of Civil Affairs, the Ministry of Health, and China Disabled Persons’ Federation. The procedures of this survey satisfied the requirements of the Statistics Law of the People’s Republic of China. All survey subjects completed the written informed consent provided by the Chinese government. All study procedures adhered to the guidelines of the Declaration of Helsinki. We obtained the data about calculating cataract disease burden from the report of the second China National Sample Survey on Disability^3^ and the China Disabled Persons’ Federation. The data provided by the China Disabled Persons’ Federation was in accordance with the relevant state regulations of statistical data.

### Diagnosis of Cataract Visual Disability

Cataract visual disability refers to uncorrectable poor vision or constriction of visual field in both eyes caused by cataracts, including blindness and low vision. The diagnostic standard of cataract visual disability is as follows^3^: (1) blindness, defined as best adjusted visual acuity in the better eye less than 0.05 or visual field radius less than 5 degrees; (2) low vision, defined as best adjusted visual acuity in the better eye less than 0.3; and (3) cataract visual disability, defined as blindness or low vision caused by lens opacity without other eye diseases. In the survey, all suspected subjects with visual disability received ocular examination by ophthalmologists, including a test for visual acuity (the Snellen chart) as well as slit-lamp and ophthalmoscopic examination of the eyelid, globe, pupillary reflex, and lens. If the subject conformed to the above diagnostic standard, they were categorized as having “visual disability due to cataract”.

### Calculating DALYs

DALYs are a summary measure of population health, combining information about the impact of premature death and disability and about other non-fatal health outcomes.^13^ DALYs include two components: years of life lost due to premature death (YLLs) and years of life lived with disabilities (YLDs). The general formula for calculating DALYs is as follows^14^:

$$\mathrm{DALYs}=\int_{x=a}^{x=a+L}DCxe^{-\beta x}e^{-r(x-a)}dx$$

which is transformed to the following formula after integration:

$$\begin{array}{ll} \mathrm{DALYs} & =-\frac{DCe^{-\beta a}}{{\left(\beta +r\right)}^{2}}\times \{e^{-(\beta +r)L}[1+(\beta +r)(L+a)]\\ & \;-[1+(\beta +r)a]\} \end{array}$$

where *D* is the disability weight, *r* is the discount rate (*r* = 0.03), *β* is the age-weighting function (*β* = 0.04), *C* is an age-weighting correction constant (*C* = 0.162 43), *a* is the age of onset, and *L* is the duration of disability or time lost owing to premature mortality.^14^

The three key variables for calculating DALYs are “the age of onset”, “the disability weight”, and “the duration of disability”. In this study, “the age of onset for cataract visual disability” was obtained from the Second China National Sample Survey on Disability. The questionnaire for disabled people of the survey included a survey item of discovery time, which was considered as “the age of onset for cataract visual disability”. The disability weight reflects the severity of the disease on a scale from 0 (perfect health) to 1 (dead). According to Global Burden of Disease (GBD) 2004, the disability weight for low vision is 0.17, and the disability weight for blindness is 0.57.^15^ The duration of disability is measured by the expectation of life at the age of onset of cataract visual disability.^14^ We used the data from the fifth national census in China (conducted by the National Bureau of Statistics of China) to work out the current life-table, from which the expectation of life at the age of onset of cataract visual disability was obtained. As the sample was all people who were vision-disabled due to cataracts, the DALYs of cataracts only include YLDs in this study.

### Measurement of ambient erythemal UVR

Erythemal UVR is commonly calculated by weighing the solar UV radiation with the erythemal action spectrum to reflect the erythemal effect of UVB radiation to the human skin. The estimated daily ambient erythemal UVR, in joules per meter squared (J/m^2^), was obtained on a 1° of latitude (89.5 S to 89.5 N) by 1° of longitude (180 W to 180 E) grid for all of China.^9^ The grid was generated from the NASA Goddard Space Flight Center Data Archive Center database of readings from the Total Ozone Mapping Spectrometer mounted on the Nimbus-7 satellite^16^ and Earth Probe satellite.^17^ We estimated the daily ambient erythemal UVR by averaging daily estimates from 1980 through 2005 and calculating the average daily ambient erythemal UVR based on the assumption that the relative distribution of annual ambient erythemal UVR was stable over time.^9^ As the NASA database did not provide a complete dataset of estimated daily ambient erythemal UVR from 1993 to 1996, data from these years were not included in this study. ArcGIS 9.2 (Esri Inc., Redlands, CA, USA) software was used for the spatial modeling of the daily ambient erythemal UVR data, and the selection function was used to obtain region-level daily ambient erythemal UVR.^9^

### Groups by latitude and altitude

To further research the relationship between DALY rates of cataracts and ambient erythemal UVR, the 31 regions were divided into groups based on latitude and altitude. The grouping method has been described in our previous study.^9^ In summary, regions with average latitude ≤30° N were allocated to latitude group 1; regions with average latitude >30° N and <40° N were allocated to the latitude group 2; regions with average latitude ≥40° N were allocated to latitude group 3 (Table 1). Regions were divided into three altitude groups based on the unique geographic feature of China, which Chinese geographers name “three-step ladder”. Regions with average altitude ≥4000 meters were allocated to the altitude group 1; regions with average altitude between 500 and 2000 meters were allocated to the altitude group 2; and regions with average altitude <500 meters were allocated to altitude group 3 (Table 1). Regions with an average altitude between 2000 meters and 4000 meters don’t exist in China.

**Table 1.** Daily ambient erythemal UVR and DALY rates of cataracts in 31 regions of China (sorted by DALYs/100 000 of all age groups)

Regions	Group		UVR (J/m^2^)	DALYs/100 000							

				All age groups					Age group		
		
	latitude	altitude		Male	Female	Agr	N-Agr	Total	0–64	65–74	≥75
Beijing	3	3	2385	25	47	50	29	36	4	178	402
Tianjin	3	3	2381	32	62	42	51	47	12	307	495
Heilongjiang	3	3	1729	48	55	63	31	51	28	323	364
Shanghai	2	3	2797	32	78	55	56	56	24	186	516
Chongqing	2	2	2553	69	84	82	55	76	29	344	610
Liaoning	3	3	2217	44	111	94	49	77	26	514	625
Shanxi	2	3	2731	43	146	103	79	94	41	535	1025
Zhejiang	1	3	2867	66	127	99	87	96	15	412	1082
Inner Mongolia	3	2	2316	84	122	104	102	103	61	456	751
Hebei	2	3	2463	72	152	130	42	112	49	604	961
Jilin	3	3	2026	74	155	150	75	114	54	639	837
Guizhou	1	2	2797	71	197	139	60	124	29	880	1782
Shaanxi	2	2	2762	120	157	158	50	133	55	723	1317
Hunan	1	3	2775	102	190	160	68	138	61	593	1033
Hubei	2	3	2721	104	207	154	112	144	61	790	1193
Shandong	2	3	2614	88	223	171	69	155	45	944	1556
Jiangsu	2	3	2708	124	205	192	73	156	32	666	1730
Ningxia	2	2	3026	179	160	188	113	164	61	1658	2027
Xinjiang	3	2	3185	90	255	210	96	169	93	1126	1494
Henan	2	3	2700	126	223	188	79	170	48	1029	2140
Jiangxi	1	3	2912	120	259	184	106	173	48	1212	1919
Fujian	1	3	3105	113	264	194	159	188	37	1133	2252
Anhui	2	3	2731	131	251	217	61	188	63	925	1766
Qinghai	2	1	4659	172	205	202	135	189	60	1397	3058
Gansu	2	2	3327	166	240	232	124	189	80	1569	1831
Guangdong	1	3	3281	135	338	230	133	203	41	1397	2408
Hainan	1	3	3913	168	347	299	113	233	42	1572	2637
Guangxi	1	3	3106	186	324	280	154	254	47	1474	2885
Sichuan	2	2	3825	228	340	290	152	255	79	1285	2067
Yunnan	1	2	3961	277	506	305	157	282	79	2271	3393
Xizang	2	1	4867	25	47	365	671	394	215	1927	5379
Total	—	—	—	105	196	177	81	150	47	854	1510

Agr, agricultural population; DALY, disability-adjusted life years; N-Agr, non-agricultural population; UVR, ultraviolet radiation.

### Statistical analysis

Spearman rank correlation analysis and linear regression analysis were performed to establish the relationship between DALY rates of cataracts and ambient erythemal UVR in China. The Kruskal-Wallis rank sum test was used to compare the DALY rates of cataracts and ambient erythemal UVR among the three latitude groups and the three altitude groups. If the results of the Kruskal-Wallis rank sum test showed statistical difference, the Wilcoxon rank sum test was used to perform pairwise comparisons. The pairs were tested in the pairwise comparisons including latitude (or altitude) group 1 and group 2; latitude (or altitude) group 1 and group 3; latitude (or altitude) group 2 and group 3. In order to avoid increase in the total probability of type 1 error, α level for pairwise comparisons was adjusted to 0.017 (0.05/3).

SPSS 12.0 statistic software (SPSS Inc., Chicago, IL, USA) was used to perform the statistical analysis. A two-sided *P* value <0.05 was considered significant.

## RESULTS

### DALY rates of cataracts and daily ambient erythemal UVR in 31 regions

As shown in Table 1, the DALY rate of cataracts was 150 DALYs/100 000 people among the whole population, with 47 DALYs/100 000 among the population <65 years old, 854 DALYs/100 000 among the population aged 65–74 years old, and 1510 DALYs/100 000 among the population ≥75 years old. The DALY rate of cataracts among females was higher than among males in all provinces except for Ningxia. The average DALY rates of cataracts among the agricultural and non-agricultural populations were 177 DALYs/100 000 and 81 DALYs/100 000, respectively.

The highest ambient erythemal UVR was recorded in Xizang (4867 J/m^2^), which also had the highest DALY rate of cataracts among the whole population (394 DALYs/100 000), among the population <65 years old (215 DALYs/100 000), and among the population ≥75 years old (5379 DALYs/100 000). Yunnan had the highest DALY rate of cataracts among the population aged 65–74 years (2271 DALYs/100 000), while the ambient erythemal UVR in Yunnan was the third highest (3961 J/m^2^). The lowest ambient erythemal UVR was recorded in Heilongjiang (1729 J/m^2^); the DALY rates of cataracts in Heilongjiang were lower than those in most of the regions among the whole population, among the population <65 years old, and among the population aged 65–74 years; the DALY rate of cataracts among a population ≥75 years old (364 DALYs/100 000) in Heilongjiang was the lowest.

Beijing had the lowest DALY rate of cataracts among the whole population (36 DALYs/100 000), among the population <65 years old (4 DALYs/100 000), and among the population aged 65–74 years (178 DALYs/100 000), as well as the second lowest DALY rate of cataracts among the population ≥75 years old (402 DALYs/100 000). Hebei, which has a similar geographical location and UVR level to Beijing, had a much higher DALY rate of cataracts than Beijing. The same situation was also observed between Shanghai and Zhejiang.

Xizang had the highest DALY rates of cataracts both in agricultural (365 DALYs/100 000) and non-agricultural populations (671 DALYs/100 000). Yunnan (305 DALYs/100 000) and Hainan (299 DALYs/100 000) had higher DALY rates of cataracts in the agricultural population than any other provinces, except for Xizang. Three provinces (Liaoning, Jilin and Heilongjiang) in northeast China had lower DALY rates of cataracts both in agricultural and non-agricultural populations. There were few differences in DALY rates of cataracts among agricultural and non-agricultural populations of Beijing, Tianjin, and Shanghai.

### Relationship between DALY rates of cataracts and ambient erythemal UVR in China

**Figure 2.**
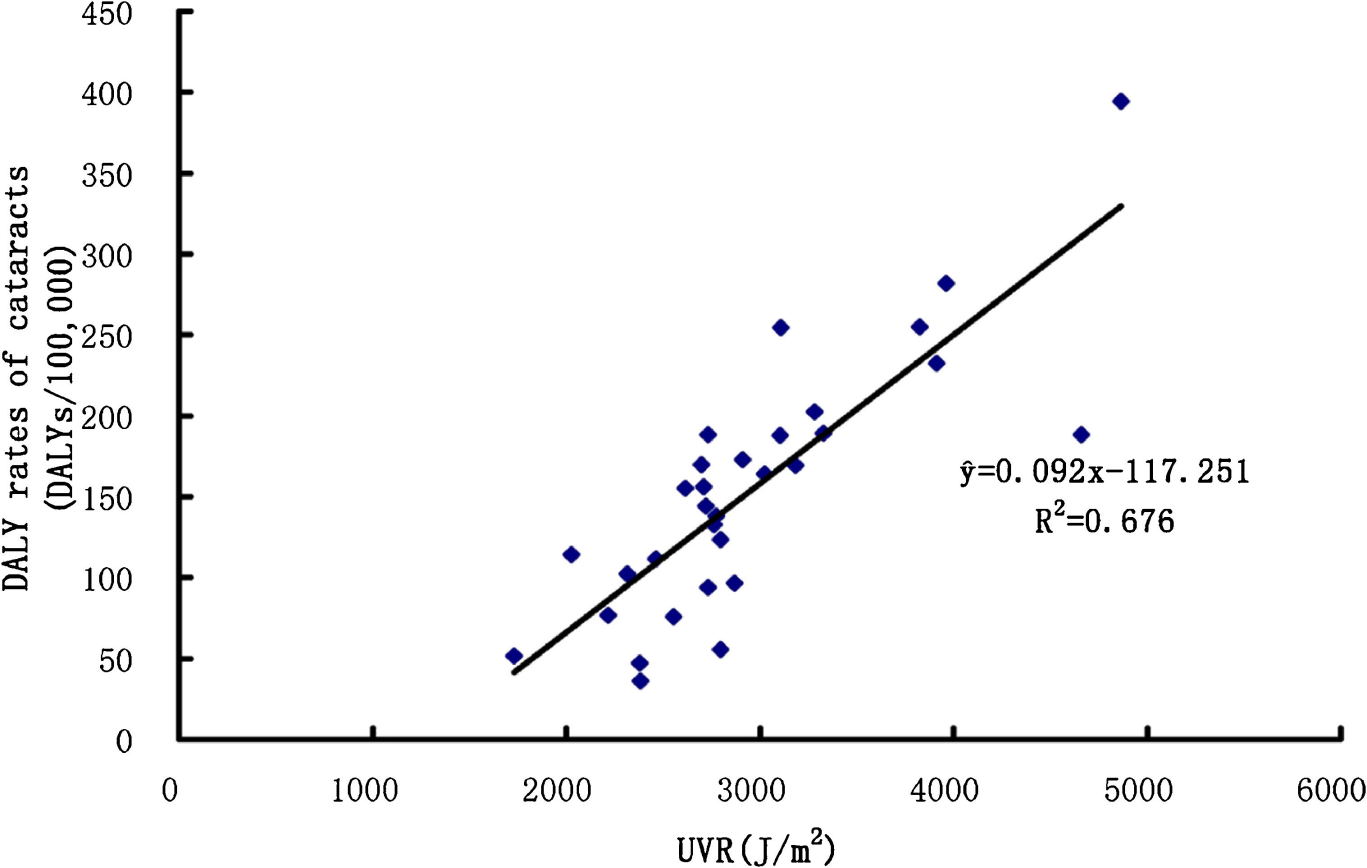
Scatter plot and regression line between the DALY rates of cataracts among the whole population (Y) and daily ambient erythemal UVR (X) in the 31 regions of China.

The DALY rate of cataracts among the whole population and in the three age groups increased with increases in the daily levels of ambient erythemal UVR. The DALY rates of cataracts among the whole population and in the three age groups were found to be associated with UVR in the Spearman rank correlation analysis (*r*_s(DALY rates of cataracts)_ = 0.811, *P* < 0.01; *r*_s(DALY rates of cataracts among a population <65 years old)_ = 0.474, *P* < 0.01; *r*_s(DALY rates of cataracts among a population aged 65–74 years old)_ = 0.796, *P* < 0.01; *r*_s(DALY rates of cataracts among a population ≥75 years old)_ = 0.830, *P* < 0.01). The linear regression equations between the DALY rates of cataracts and UVR were $\hat{y}$ = 0.092*x* − 117.251 (*R*^2^ = 0.676) among the whole population, $\hat{y}$ = 0.034*x* − 49.418 (*R*^2^ = 0.423) among the population <65 years old, $\hat{y}$ = 0.607*x* − 852.367 (*R*^2^ = 0.617) among the population aged 65–74 years, and $\hat{y}$ = 1.342*x* − 2295.355 (*R*^2^ = 0.758) among the population ≥75 years old. In the equations, *x* was the amount of UVR, $\hat{y}$ was the DALY rates of cataracts, and *R*^2^ was the coefficient of determination. As the four regression equations had similar changing trends, we only showed the scatter plot and regression line between the DALY rates of cataracts among the whole population and daily ambient erythemal UVR in the 31 regions of China in Figure 2.

### Influence of latitude and altitude on daily ambient erythemal UVR and DALY rates of cataracts

The medians of UVR in the 3 latitude groups were significantly different (*P* = 0.002), as were the medians of DALY rates among the whole population (*P* = 0.009), among the population aged 65–74 years (*P* = 0.018), and among the population ≥75 years old (*P* = 0.002). The medians of DALY rates among the population <65 years old had no statistical difference among 3 latitude groups. The median DALY rate among the whole population in latitude group 3 was 111 DALYs/100 000 lower than that of the latitude group 1 (*P* = 0.003) and 79 DALYs/100 000 lower than that of latitude group 2 (*P* = 0.014). Among the population aged 65–74 years, the median DALY rate of latitude group 3 was 756 DALYs/100 000 lower than that of latitude group 1 (*P* = 0.008). Among the population ≥75 years old, the median DALY rate of latitude group 3 was 1627 DALYs/100 000 lower than that of latitude group 1 (*P* = 0.001) and 1105 DALYs/100 000 lower than that of latitude group 2 (*P* = 0.003; Table 2).

**Table 2.** Daily ambient erythemal UVR and DALY rates of cataracts in latitude groups

Latitude group^a^	UVR (J/m^2^) Median	*P*	DALYs/100 000 Median									

			Total	*P*	0–64^e^	65–74	*P*	≥75	*P*	Agr	*P*	N-Agr^e^
1	3105	0.073^b^	188	0.290^b^	42	1212	0.347^b^	2252	0.138^b^	217	0.411^b^	106
2	2731	0.002^c^	156	0.014^c^	55	925	0.021^c^	1730	0.003^c^	188	0.026^c^	79
3	2316	0.005^d^	77	0.003^d^	28	456	0.008^d^	625	0.001^d^	94	0.012^d^	51

^a^Group 1: average latitude ≤30°N; group 2: average latitude 30°N–40°N; group 3: average latitude ≥40°N.

Agr, agricultural population; DALY, disability-adjusted life years; N-Agr, non-agricultural population; UVR, ultraviolet radiation.

^b^Pairwise comparison between group 1 and 2.

^c^Pairwise comparison between group 2 and 3.

^d^Pairwise comparison between group 1 and 3.

^e^No statistical significance among the 3 latitude groups; no pairwise comparison required.

The medians of the DALY rates of the agricultural population (*P* = 0.025) were significantly different among the 3 latitude groups. The average disease burden of the agricultural population in latitude group 1 was 217 DALYs/100 000, which was more than two times that of latitude group 3 (94 DALYs/100 000; *P* = 0.012). However, the median DALY rates of the non-agricultural population did not differ among the 3 latitude groups (Table 2).

The medians of UVR differed significantly among the 3 altitude groups (*P* = 0.018), and the median of UVR of altitude group 1 was 2048 J/m^2^ higher than that of altitude group 3 (*P* = 0.009). Although the medians of DALY rates of the whole population, the three age groups, the agricultural population, and the non-agricultural population in the 3 altitude groups showed obvious respective gradients, there was still no statistical difference among the 3 altitude groups (Table 3).

**Table 3.** Daily ambient erythemal UVR and DALY rates of cataracts in altitude groups

Altitude group	Average altitude	UVR (J/m^2^) Median	*P*	DALYs/100 000 Median					

				Total^d^	0–64^d^	65–74^d^	≥75^d^	Agr^d^	N-Agr^d^
1	≥4000	4763	0.036^a^	291	137.5	1662	4218.5	298.5	397.5
2	500–2000	3026	0.085^b^	164	61	1126	1782	188	102
3	≤500	2715	0.009^c^	141	42	652	1137.5	157	74

Agr, agricultural population; DALY, disability-adjusted life years; N-Agr, non-agricultural population; UVR, ultraviolet radiation.

^a^Pairwise comparison between group 1 and 2.

^b^Pairwise comparison between group 2 and 3.

^c^Pairwise comparison between group 1 and 3.

^d^No statistical significance among the 3 altitude groups; no pairwise comparison required.

## DISCUSSION

The present study demonstrated that the DALY rate of cataracts among the whole population was 150 DALYs/100 000, which was lower than the rate (268 DALYs/100 000) estimated by GBD 2004.^18^ The differences were most likely due to discrepancies between the data sources and methods for calculating DALYs of the two sets of results. The WHO used the prevalence of cataracts and DisMod II software to calculate DALYs, a method that uses many estimates. In this study, the disease burden of cataracts in China was calculated based on data from the Second China National Sample Survey on Disability, which was a nationwide sample survey organized by the Chinese government. The basic data provided in this survey were able to ensure the accuracy of DALY calculations.

The geographic characteristics of China result in a clear gradient in UVR levels. Xizang, the region with the highest average altitude in China, had the highest UVR (4867 J/m^2^) and highest DALY rates among the whole sample (394 DALYs/100 000), among people <65 years old (215 DALYs/100 000) and among people ≥75 years old (5379 DALYs/100 000). Hainan, the southernmost region in China with the lowest average latitude, also had very high UVR (3913 J/m^2^) and DALY rates of cataracts among the whole population (233 DALYs/100 000), the population aged 65–74 years (1572 DALYs/100 000), and the population ≥75 years old (2637 DALYs/100 000). Conversely, Heilongjiang, the northernmost region in China with low altitudes, had the lowest UVR and lower DALY rates of cataracts among the whole population and in the three age groups. These results showed that the DALY rates of cataracts in regions with higher ambient erythemal UVR were higher than those in regions with lower ambient erythemal UVR.

However, the DALY rates of cataracts in municipalities directly under the Central Government had remarkable differences from the adjoining regions, even though the UVR in the adjoining regions was very close to that in the municipalities. For example, Beijing and Shanghai had lower DALY rates compared to Hebei and Zhejiang, which are geographically similar to Beijing and Shanghai, respectively. The findings were thus consistent with the findings of our previous study that the more urbanized regions had a lower disability prevalence of cataracts.^9^ Beijing and Shanghai had higher levels of urbanization, and so a majority of citizens spent more time working indoors, leading to a lower level of actual UVR exposure than citizens in other regions. In addition, Beijing and Shanghai possess better cataract surgical services due to their better economic development. People with cataracts living in these municipalities can be treated in time to reduce their likelihood of visual disability.

In general, we found that the DALY rates of cataracts among the whole population and in the three age groups had a positive correlation with UVR exposure in the 31 regions. In our previous study, the disability prevalence of cataracts was also positively associated with UVR exposure in the 31 regions.^9^ We constructed regression equations to estimate the DALY rates of cataracts under certain UVR exposures, demonstrating that an increase in the average UVR by 1000 J/m^2^ was associated with an increase in the DALY rates of cataracts of 92 DALYs/100 000 among the whole population, 34 DALYs/100 000 among the population <65 years old, 607 DALYs/100 000 among the population aged 65–74 years, and 1342 DALYs/100 000 among the population ≥75 years old. The results showed that people living in regions with higher UVR exposure and the elderly bore heavier disease burden of cataracts.

Latitude and altitude are closely related with the level of UVR exposure. In our previous study, we found that the disability prevalence of cataracts was significantly higher in low- and middle-latitude regions than in high-latitude regions, but not significantly different in the three altitude groups.^9^ The analysis among latitude groups in this study also showed that higher DALY rates of cataracts were observed in regions with higher UVR. Data from the research of Javitt and Taylor showed that latitude had some correlation with cataract prevalence and that the probability of cataract surgery in the U.S. increased by 3% for each 1° decrease in latitude.^19^ The DALY rates of cataracts among the whole population and in the three age groups were not significantly different among the three altitude groups in our study, which could be the result of the smaller sample size in altitude group 1.

The actual level of UVR exposure is also correlated with hours spent outdoors. A case-control study in Xizang showed greater risk of senile cataracts in those who worked outside for more than 6 hours a day.^20^ In our study, we noted a remarkable difference in disease burden of cataracts between the agricultural and non-agricultural populations. The disease burden in the agricultural population was almost three times that in the non-agricultural population. Compared with the non-agricultural population, the agricultural population spent more time working outdoors, resulting in a high level of UVR exposure, a higher prevalence of cataracts, and a heavier disease burden of cataract visual disability. The disease burden of cataracts among the agricultural population also varied geographically, with greater burden in regions with low latitudes than in regions with high latitudes. Regions with low latitudes have subtropical climates, in which crops are ripe two to three times a year, requiring workers to spend more time working outdoors. In contrast, in the northeastern regions, temperatures are lower and the crop growth period only lasts for 4 to 7 months, which reduces the amount of time laborers spend outdoors and subsequently their actual UVR exposure. Beijing, Tianjin, and Shanghai had the lowest disease burdens of cataract visual disability among the 31 regions, which was associated with the high level of urbanization in these areas. Only a small portion of the population is engaged in outdoor labor in these cities, which results in a lower average level of UVR exposure and lower disease burden of cataract visual disability. In addition, other factors also enlarge the gap between urban and rural areas in disease burden due to cataract visual disability, such as economic levels and capacity of ophthalmological services.

According to previous studies, cataracts are primarily an age-related disorder that are more closely associated with UVR exposure among the elderly than among younger people.^20^^,^^21^ In this study, the number of cataract-disabled people aged ≥65 years accounted for 83.8% of all cataract-disabled people and 60% of the group of people ≥65 years old were aged ≥75 years. The prevalence of cataract visual disability among people aged ≥65 years was 6.05%. Estimations based on the number of people aged 65 years in the sixth census^22^ indicated that there were approximately 2.89 million cataract visually disabled people aged ≥65 years old in China. With the growing and aging population in China, the number of people with cataracts, especially those with senile cataracts, will only increase further. In this study, we estimated the DALY rates of cataracts among the population aged 65–74 years and among the population ≥75 years old, whose DALY rates of cataracts were much higher than those among the population <65 years old. We also analyzed the relationship between the DALY rates of cataracts and ambient erythemal UVR in the three age groups, which demonstrated that the change in the amount of UVR exposure had greater influence on the disease burden among the population aged 65–74 years and among the population ≥75 years old.

However, there were several limitations to this study. As people are not distributed evenly in a specific region, it was difficult for us to detect correspondence between demographic data and UVR level. So we had to make a rough estimation by using the average ambient erythemal UVR in each region to stand for the ambient UVR. The actual amount of personal UVR exposure may not be represented by the ambient erythemal UVR, as it is easily affected by people’s lifestyles. In this study, the differences in UVR exposure were only reflected indirectly by assessing agricultural and non-agricultural populations. There is a general consensus that UVR exposure is more closely related to cortical cataracts than nuclear cataracts.^4^^,^^7^^,^^23^^,^^24^ However, other researchers have detected a possible connection between sunlight and nuclear cataracts.^6^^,^^25^ A high incidence of nuclear cataracts has been reported for certain regions having high UVR exposure.^26^ The Second China National Sample Survey on Disability did not request details regarding the types of cataracts, so we could not distinguish between different types of cataracts in this study. Other studies indicated that high socio-economic level and adequate cataract surgical coverage could decrease the prevalence of cataract disability,^27^^,^^28^ while smoking increases the risk of cataract development.^29^^,^^30^ These factors, which were not assessed in the present study, may also influence the disease burden of cataracts.

Notwithstanding these limitations, however, the findings of this study provide scientific evidence to support the relationship between the level of UVR exposure and disease burden of cataracts. We have reason to believe that increased exposure to UVR increases the disease burden of cataracts in the whole population, especially in the elderly and among the agricultural population. These findings will help to plan future strategies to prevent the development of cataract and cataract visual disability and improve the quality of life in cataract patients.
