# The Relationship Between Disability-Adjusted Life Years of Cataracts and Ambient Erythemal Ultraviolet Radiation in China

**DOI:** 10.2188/jea.JE20140208

**Published:** 2015-02-05

**Authors:** Min Zhu, Jiaming Yu, Qian Gao, Yang Wang, Liwen Hu, Yang Zheng, Fang Wang, Yang Liu

In this article, we found errors in the DALYs/100 000 of men and women in 18 regions. We therefore corrected Table 1 (only the revised data are presented). In addition, the main text from the eighth to the ninth line of the Results section should be amended as follows: “The DALY rate of cataracts among females was higher than that among males in all provinces except for Xinjiang.” Furthermore, a period should be added after the first “UVR” in the tenth line of the Abstract. The authors regret these errors.

**Table 1.** Daily ambient erythemal UVR and DALY rates of cataracts in 31 regions of China (sorted by DALYs/100 000 of all age groups) (corrected part only)

Regions	Group		UVR (J/m^2^)	DALYs/100 000							

				All age groups					Age group (years)		
		
	latitude	altitude		Male	Female	Agr	N-Agr	Total	0–64	65–74	≥75
Guizhou	1	2	2797	86	164	139	60	124	29	880	1782
Shaanxi	2	2	2762	71	197	158	50	133	55	723	1317
Hunan	1	3	2775	120	157	160	68	138	61	593	1033
Hubei	2	3	2721	102	190	154	112	144	61	790	1193
Shandong	2	3	2614	104	207	171	69	155	45	944	1556
Jiangsu	2	3	2708	88	223	192	73	156	32	666	1730
Ningxia	2	2	3026	124	205	188	113	164	61	1658	2027
Xinjiang	3	2	3185	179	160	210	96	169	93	1126	1494
Henan	2	3	2700	90	255	188	79	170	48	1029	2140
Jiangxi	1	3	2912	126	223	184	106	173	48	1212	1919
Anhui	2	3	2731	120	259	217	61	188	63	925	1766
Gansu	2	2	3327	131	251	232	124	189	80	1569	1831
Guangdong	1	3	3281	166	240	230	133	203	41	1397	2408
Hainan	1	3	3913	135	338	299	113	233	42	1572	2637
Guangxi	1	3	3106	168	347	280	154	254	47	1474	2885
Sichuan	2	2	3825	186	324	290	152	255	79	1285	2067
Yunnan	1	2	3961	228	340	305	157	282	79	2271	3393
Xizang	2	1	4867	277	506	365	671	394	215	1927	5379

Agr, agricultural population; DALY, disability-adjusted life years; N-Agr, non-agricultural population; UVR, ultraviolet radiation.

